# Donor human milk versus infant formula for low-risk infants: a systematic review

**DOI:** 10.1038/s41390-024-03309-x

**Published:** 2024-06-06

**Authors:** Thomas McClintock, Catherine Fiddes, Shalee Harris, Nicholas Embleton, Luling Lin, Frank H. Bloomfield, Mariana Muelbert

**Affiliations:** 1 Liggins Institute, University of Auckland, Auckland, New Zealand; 2 Newcastle Upon Tyne Hospitals NHS Foundation Trust, Newcastle upon Tyne, UK; 3 Population Health Sciences Institute, Newcastle University, Newcastle upon Tyne, UK

## Abstract

**Background:**

There is an increasing acceptance and use of donor human milk (DHM) in healthy infants. This review investigates the benefits and risks of mothers’ own milk (MOM) supplementation with DHM compared to infant formula (IF) in moderate-late preterm (MLP) and early term (ET) infants.

**Methods:**

MEDLINE, EMBASE, CINAHL, Scopus, Cochrane CENTRAL and clinical trial registries were searched for studies published up to September 2023. The primary outcome was rates of exclusive breastfeeding (EBF). Certainty of evidence was assessed using GRADE framework. RoB1 and EPHPP were used to assess risk of bias for controlled trials and observational studies, respectively.

**Results:**

Eleven studies involving total of 10,147 infants and six ongoing trials were identified. Studies were of low quality, and the certainty of evidence was assessed as very low. Three studies suggested benefits of DHM compared to IF on EBF at discharge, while two suggested no difference. No clear effect was observed on EBF duration, any breastfeeding, hypoglycemia and morbidity. No health risks were reported.

**Conclusion:**

The effect of supplementing MOM with DHM instead of IF on EBF and other health outcomes is unclear. High-quality studies are required to determine the potential benefits or risks of DHM supplementation in this population.

**Impact:**

- We identified 11 relevant studies reporting on supplementation of mothers’ own milk (MOM) with donor human milk (DHM) compared to infant formula (IF). Studies were of low quality, had heterogeneous outcome definitions and were geographically limited; all except two were observational studies.
- Limited evidence showed no clear difference on rates of exclusive breastfeeding and other health outcomes. No potential risks were reported.
- The increasing acceptance and use of DHM in healthy infants highlights the need for future high-quality studies.

## Introduction

Infants born between 32 and 36 completed weeks’ gestation are considered moderate to late preterm (MLP). They are the largest population of preterm infants, representing 85% of preterm births worldwide.^1^ Due to their relative metabolic and physiological immaturity, MLP infants have increased morbidity compared to full-term (FT) infants born between 39 and 41 complete weeks’ gestation.^2,3^ Infants born between 37 and 38 complete weeks’ gestation are considered early term (ET), accounting for approximately five times as many births as MLP infants. ET infants are also at increased risk of morbidity compared to FT infants.^3,4^ The higher morbidity associated with ET and MLP birth appears to influence lifelong health.^3,5–7^ Thus, MLP and ET infants pose a significant economic burden to healthcare systems worldwide.^3^

The benefits of breastfeeding for mothers and infants are well recognised. Breastfeeding reduces the rates of many childhood morbidities, such as otitis media, lower respiratory tract infections, and severe diarrhoea.^8^ Nevertheless, MLP and ET infants have poorer rates of breastfeeding initiation and duration than FT infants^9^ and are more likely to require supplemental milk feeding due to factors such as hypoglycemia or delayed onset of secretory activation.^10^ Evidence to support nutritional guidelines for MLP and ET infants is lacking; resulting in significant variation in practice.^10–12^

Most international guidelines strongly endorse mothers’ own milk (MOM) as the preferred feeding for MLP infants and emphasise that mothers should receive qualified, extended lactation support.^8,10^ There is limited evidence on the best form of supplementation of MOM for this population, with some studies suggesting that a limited amount of formula may actually facilitate ongoing breastfeeding in healthy term infants,^13,14^ while others suggest that even short exposure to formula supplementation may be detrimental to breastfeeding outcomes.^13,15,16^

The use of donor human milk (DHM) for feeding very preterm and low birthweight infants has been extensively studied and is associated with decreased risk of necrotising enterocolitis (NEC) and feed intolerance but slower growth compared to preterm formula.^17–21^ Whether DHM as a temporary supplement when MOM is insufficient may also confer health benefits for MLP and ET infants over the use of infant formula (IF) is less clear and has not been systematically appraised.^17^ This systematic review aims to investigate the risks and benefits of using DHM compared to IF for supplementation of MOM in MLP and ET infants.

## Methods

This review was conducted according to the Cochrane Handbook for Systematic Reviews of Interventions,^19^ reported following the Preferred Reporting Items for Systematic Reviews and Meta-Analyses (PRISMA) guidelines^20^ and registered prospectively in PROSPERO (registration number CRD42022329890).

We systematically searched MEDLINE via Ovid, EMBASE, CINAHL Complete, Scopus, Cochrane Central Register of Controlled Trials (CENTRAL), ClinicalTrials.gov, WHO’s International Trial registry and platform, and the Australian New Zealand Clinical Trials Registry for publications published up to 22 September 2023. Studies were eligible for inclusion if they: (1) included MLP or ET infants with a birth weight of > 1500 g, requiring supplementation of MOM with DHM or IF; (2) involved any of the outcomes of interest, and (3) were a randomized or observational study.

Primary outcome was the rate of exclusive breastfeeding (EBF) at discharge or beyond (as defined by investigators). Other outcomes of interest included:

- any breastfeeding at discharge or beyond (receiving any breastmilk, or as defined by investigators);
- growth (weight, length, head circumference and z-scores, growth velocity, body composition, body mass index at any time point, as defined by investigators);
- incidence of feed intolerance during hospitalisation (resulting in cessation or reduction of feeds, or as defined by investigators);
- duration of nutritional support (parenteral nutrition and/or enteral feeding), measured in days;
- duration of hospital stay, measured in days;
- incidence of infection during hospital stay (positive culture in a normally sterile bodily fluid, or as defined by investigators);
- incidence of NEC Bell’s Stage 2^21^ or more;
- incidence of gastroenteritis during hospitalisation (gastrointestinal infection with diarrhea and/or dehydration);
- incidence of hypoglycemia (blood glucose < 2.6 mmol/L, or as defined by investigators);
- incidence of neonatal morbidity (incidence of re-hospitalisation, respiratory or gastrointestinal infection in the first month of life);
- incidence of childhood morbidity (incidence of re-hospitalisation, overweight and obesity, respiratory or gastrointestinal infection, otitis or allergy);
- neurodevelopmental outcome during childhood (Age and Stages Questionnaire (ASQ) total score, Bayley Scales of Infant and Toddler Development, or as defined by investigators), and
- health economic analysis (any cost analysis associated with supplementation of MOM, or as defined by investigators).

Any breastfeeding at discharge or beyond was not a pre-specified outcome in the protocol but was included as an additional outcome of interest as it was commonly reported by eligible studies.

Studies were excluded if they included infants with congenital abnormalities or genetic/ metabolic disorders. No restrictions were applied for date, language or country of publication. A search strategy with key terms in English was developed. (Supplementary table 1).

Search results were imported into Covidence systematic review software (2023, Veritas Health Innovation, Melbourne), and duplicates removed. Two investigators (T.M. and C.F.) independently screened titles and abstracts and excluded irrelevant studies, then reviewed the full text of reports for compliance with the eligibility criteria and extracted data into a pre-specified data extraction form. Where data were presented exclusively in the form of charts, we utilised a web tool to extract necessary information.^22^

The risk of bias of randomized controlled trials (RCT) was evaluated using the Cochrane Risk of Bias Tool 1 (RoB1).^23^ and the quality of observational studies was assessed using the Quality Assessment Tool for Quantitative Studies developed by the Effective Public Healthcare Panacea Project (EPHPP).^24^ The Risk-of-bias VISualization (robvis) tool.^25^ was used to generate a figure with the quality of observational studies.

The certainty of evidence of included studies was evaluated using the Grading of Recommendations Assessment, Development and Evaluation (GRADE) tool.^26^ for the following pre-specified outcomes: EBF at discharge or beyond; growth; duration of hospital stay; incidence of feed intolerance; incidence of infection; gastroenteritis; and hypoglycemia. These outcomes were selected during the protocol stage and judged by the authors as critical outcomes for informing clinical recommendations.

All steps were conducted independently by two investigators (T.M. and C.F.) and disagreements were resolved by discussion with a third reviewer (M.M.). We intended to perform meta-analysis, but this was not possible because of the heterogeneity of included studies.

## Results

### Results of search strategy

**Fig. 1:**
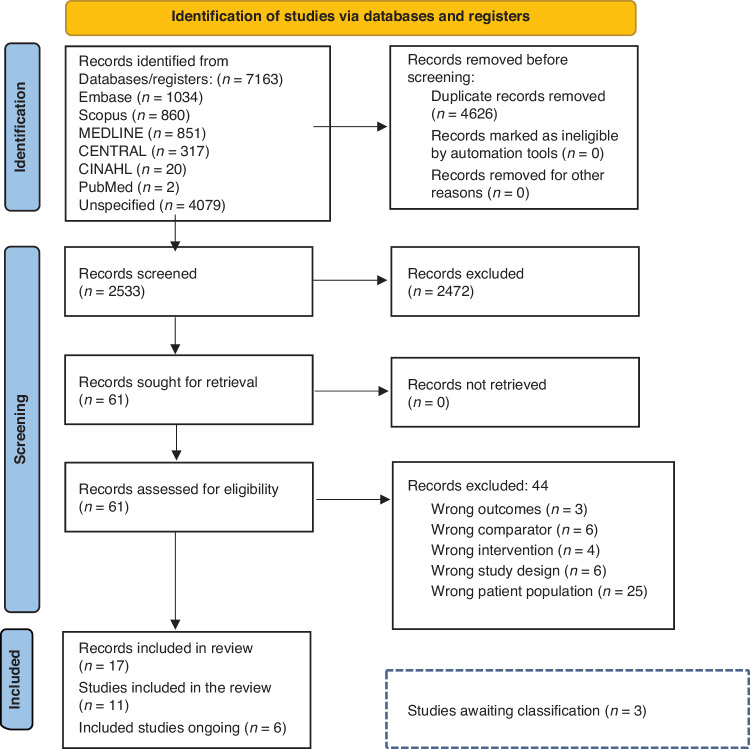
Flow diagram of study selection. Flow diagram of study identification and selection via databases and registers.

Initially, 7163 records were identified. After duplicate removal, 2533 titles and abstracts were screened, of which 61 records were included in full-text screening. Of these, 44 did not meet the inclusion criteria and were excluded; 17 records met the inclusion criteria (Fig. 1), of which 11 studies were included in the review (Table 1) and six studies were identified as ongoing. The characteristics of the six ongoing studies, all RCTs, are described in Supplementary table 2.

**Table 1:** Characteristics of included studies.

Author/Year	Setting and date	Participants, *N*	Intervention/ exposure (DHM), *n*	Comparison, *n*	Outcomes of interest
*Randomised controlled trials*					
Saarinen 1999^#^	Finland: 3 maternity hospitals’ delivery wards Helsinki region08/1994-11/1995	Inclusion criteria: Healthy, full-term infants (no stratification by gestational age). Exclusion criteria: not stated. Reason for supplementation: insufficient breastmilk production. *N* = 6141*	Supplementation with pasteurised DHM in hospital. *n* = 1844	Supplementation either with cow’s milk formula or whey hydrolysate formula. Cow’s milk formula *n* = 1758 Whey hydrolysate formula *n* = 1715 Reference group (breastmilk fed) *n* = 824	Incidence of cow’s milk allergy.
Pithia 2023	USA: 2 NICUs at the University of California, Los Angeles, California12/2020-02/2022	Inclusion criteria: Infants with GA > 34 weeks, <48 hours of age, NICU admission, predicted NICU stay > 72 h, and a mother who intended to breastfeed. Exclusion criteria: genetic syndromes affecting growth or feeding, mechanical ventilation, common breastfeeding contraindications, major congenital anomalies. Reason for supplementation: when MOM not available. *N* = 32	Supplementation of MOM with DHM when MOM was unavailable while in NICU, ending at seven days of life or if the subject was transferred to the well-baby nursery or discharged from NICU before seven days of life. *n* = 16	Supplementation of MOM with IF when MOM was unavailable while in NICU, ending at seven days of life or if the subject was transferred to the well-baby nursery or discharged from NICU before seven days of life. *n* = 16	Rate of exclusive breastfeeding at 6-8 weeks chronological age, growth (weight, length and head circumference) at NICU discharge and 6–8 weeks chronological age.
*Prospective cohort studies*					
Gray 2022^#^	USA: Newborn nursery at a Rural academic hospital, Vermont 06/2016-06/2018	Inclusion criteria: Infants with GA ≥ 35 weeks, admitted to newborn nursery and parents indicated intention to exclusively breastfeed. Exclusion criteria: NICU admission more than 4 hours, parental intent to formula or bottle feed newborn. Reason for supplementation: newborn indications (hypoglycemia, excessive weight loss, feeding difficulties), parental request, or parental indications (delayed lactogenesis). *N* = 307	Post implementation of supplementation with DHM (12/2016-06/2018). *n* = 162	Supplementation with formula both pre and post implementation of DHM supplementation. Pre-DHM *n* = 63 Post-DHM *n* = 82	Breastfeeding or feeding pumped milk at 2 and 6 months of age.
Ponnapakkam 2021^#^	USA: Newborn nursery at Brooke Army Medical Centre, San Antonio, Texas2018-2019	Inclusion criteria: Infants with GA > 35 weeks, otherwise well, met criteria for initiation of hypoglycemia bundle based on risk factors for neonatal hypoglycaemia. Exclusion criteria: Neonates without risk factors for neonatal hypoglycemia, requiring respiratory support, requiring transfer to NICU for any other diagnosis than neonatal hypoglycemia. Reason for supplementation: hypoglycemia (point of care glucose test ≤ 44 mg/dL). *N* = 1223	Implementation of new guidelines for neonatal hypoglycaemia, with pasteurised DHM as an option for supplementation. *n* = 634	Using existing treatment algorithm for infants at risk for neonatal hypoglycaemia consisting of supplemental feeding using infant formula. *n* = 589	NICU admission for hypoglycemia, rate of exclusive breastfeeding at discharge amongst families that desired it at admission, mean change in blood glucose following treatment for hypoglycemia.
Riley 2021	USA: Postpartum unit at an academic, urban tertiary care hospital, Boston, Massachusetts2016-2017	Inclusion criteria: Healthy singleton infants with GA > 36 weeks, requiring nutritional supplementation. Exclusion criteria: Families intending to feed their infant formula without medical indication, infants admitted to NICU. Reason for supplementation: breastfeeding challenges. *N* = 39	Supplementation with pasteurised DHM in hospital. *n* = 15	Supplementation with infant formula in hospital. *n* = 24	Breastfeeding self-efficacy scale scores, infant intention scale scores during hospitalisation and at 1 month, timing of lactogenesis II, feeding outcomes during hospitalisation and at 1 month.
*Retrospective cohort studies*					
Alissa 2021^#^	USA: Mother-baby unit at University of Florida Health, Jacksonville, Florida10/2011-10/2019	Unknown (abstract) Reason for supplementation: not stated. *N* = 122	After implementation of donor human milk supplementation in Mother-Baby unit. *n* = 49	Before implementation of donor human milk supplementation in mother-baby unit. *n* = 73	Yearly exclusive breastfeeding rates at time of discharge.
Mannel 2017	USA: Mother-baby unit of a tertiary care teaching hospital, Oklahoma11/2014-10/2016	Inclusion criteria: Infants with GA 35 0/7 to 36 6/7 weeks admitted to mother-baby unit. Exclusion criteria: Infants admitted or transferred to NICU, death from anencephaly, multiple gestations, maternal complications or illness that led to separation, and infants entered into state custody. Reason for supplementation: maternal request, physician order for medical indication, or other. N=156	Supplementation with pasteurised DHM or expressed human milk in hospital. *n* = 20	Formula feeding only or supplementation of mothers’ milk with any formula in hospital. *n* = 136	Length of hospital stay, feeding status at discharge, medical complications (hypoglycemia, hyperbilirubinemia, difficulty breastfeeding).
Merjaneh 2020	USA: Newborn nursery at University of Florida Health, Jacksonville, Florida 06-09/2015-01-10/2016	Inclusion criteria: Admission to newborn nursery, mother’s intent to exclusively breastfeed, need for nutritional supplements indicated. Exclusion criteria: Infants transferred to NICU. Reason for supplementation: medical indication (hypoglycemia, hyperbilirubinemia, and > 8% weight loss at 40 h of life). *N* = 72	Supplementation with DHM in hospital. *n* = 33	Supplementation with formula in hospital. *n* = 39	Exclusive breastfeeding at 6 months, duration of any breastfeeding, time of solid food introduction.
Sen 2020^#^	USA: Boston Medical Center & Brigham and Women’s Hospital, Boston, Massachusetts 02/2016-12/2019	Inclusion criteria: Infants with GA ≥ 35 weeks who received dextrose gel within the first 48 hours for neonatal hypoglycemia. Exclusion criteria: Infants who did not receive feeding or received a combination of different feeding types with first dextrose gel. Reason for supplementation: hypoglycaemia as per hospital protocol (point of care blood glucose < 40 mg/dL). *N* = 66	Feeding with DHM with first dextrose gel. *n* = 33	Feeding with formula with first dextrose gel. *n* = 33	Median change in blood glucose after first dextrose gel, odds of needing second dextrose gel, odds of recurrent neonatal hypoglycemia.
*Cohort (unclear whether prospective or retrospective)*					
Heizelman 2020^#^	USA (years unclear)	Unclear (abstract). Reason for supplementation: Hypoglycemia (blood glucose threshold not reported). *N* = 358	Supplementation with DHM. *n* = 110	Supplementation with formula. *n* = 248	ICU admission rate, total number of dextrose gel doses required, ability to resolve hypoglycemia with one gel dose, feeding intention at discharge.
*Cross sectional studies*					
Ikonen 2023^#^	Finland 10/2019	Inclusion criteria: Singleton, full-term infants with GA ≥ 37 weeks, normal weight between 2500-4499 g. Exclusion criteria: Twins, preterm, low birth weight, fetal macrosomia infants. Reason for supplementation: not stated. *N* = 1631	In-hospital supplementation with DHM. *n* = 1237	In-hospital supplementation with formula. *n* = 394	Exclusive breastfeeding at 0–1 months, 2–3 months, 4–5 months. Any breastfeeding between 0 and 1 month, 2–3 months, 4–5 months and 6–7 months.

*GA* Gestational age, *DHM* donor human milk, *NICU* Neonatal intensive care unit, *MOM* Mothers’ own milk.*Study includes other participants beyond groups of interest.

^#^additional information requested by email.

Three studies were marked as awaiting classification as we were unable to determine the characteristics of the included population and attempts to contact authors for clarification were unsuccessful.^27–29^

### Characteristics of included studies

The characteristics of the included studies are described in Table 1. Included studies were published between 1999 and 2023 and involved 10,147 infants, with sample sizes ranging from 32 to 6209. Two RCTs were identified, one from Finland,^30^ and the second a pilot study from the USA.^31^ The remaining studies were eight cohort studies from the USA.^15,32–38^ and one cross-sectional study conducted in Finland.^39^ Only abstracts were available for two studies.^32,35^

Most of the included studies were conducted in newborn nurseries or postnatal wards.^15,30,33,35–38^ In nine studies, supplementation of MOM with DHM was directly compared with IF supplementation.^15,30–34,37–39^ Two studies compared groups before and after protocol changes implementing in-hospital supplementation with DHM.^35,36^ The gestational age (GA) of participants ranged from 30 weeks to full-term.

### Effects of the intervention

Twelve outcomes of interest were reported. Heterogeneity in study design and outcome definitions prevented us from performing meta-analysis. Thus, a summary of studies indicating a potential benefit, an unclear effect, or a possible harm of the use of DHM compared to IF for supplementation of MOM is presented in Table 2.

**Table 2:** Direction of findings.

Outcome	Direction of association		
	Studies showing possible benefit of DHM supplementation	Studies showing no clear difference of DHM supplementation	Studies showing possible harm of DHM supplementation
*Breastfeeding*			
Rate of exclusive breastfeeding at discharge.	Alissa 2021* Mannel 2017	Heizelman 2020* Ponnapakkam 2021 Merjaneh 2020	-
Rate of exclusive breastfeeding after discharge	Ikonen 2023^†^ Merjaneh 2020	Ikonen 2023^†^ Pithia 2023	-
Any breastfeeding at discharge	Mannel 2017	-	-
Any breastfeeding after discharge	Gray 2022 Ikonen 2023^†^	Riley 2021 Ikonen 2023^†^ Pithia 2023	-
*Hypoglycaemia*			
Symptomatic hypoglycaemia	-	Ponnapakkam 2021	-
Hypoglycaemia resolution with 1 dose of dextrose gel	-	Heizelman 2020*	-
Number of additional treatments to resolve hypoglycaemia (oral dextrose gel + supplemental feed)	-	Heizelman 2020*	-
Time to final hypoglycaemic episode	-	Ponnapakkam 2021	-
Average change in blood glucose	Ponnapakkam 2021	Sen 2020	-
*Growth*			
Mean weight at discharge/end of intervention	-	Pithia 2023	-
Mean length at discharge/end of intervention	-	Pithia 2023	-
Mean head circumference at discharge/end of intervention	-	Pithia 2023	-
Mean weight at 6-8 weeks chronological age	-	Pithia 2023	-
Mean length at 6-8 weeks chronological age	-	Pithia 2023	-
Mean head circumference at 6-8 weeks chronological age	-	-	Pithia 2023
*Neonatal Morbidity*			
NICU admission	-	Heizelman 2020* Ponnapakkam 2021	-
Length of hospital stay	-	Mannel 2017	-
*Childhood Morbidity*			
Risk of Cows milk allergy	-	Saarinen 1999	-

*DHM* Donor human milk, *NICU* Neonatal intensive care unit.

*only abstract available, -No study falls into this category. ^†^outcome measured at different timepoints but only statistically significant at 4–5 months.

#### Primary outcome

##### Exclusive breastfeeding at hospital discharge

Five studies reported the effects of DHM supplementation on rates of EBF at hospital discharge.^15,32,33,35,36^ One retrospective cohort study reported that 100% of infants supplemented with human milk in hospital (either expressed breastmilk or DHM) were EBF at hospital discharge, whereas no infants supplemented with IF in hospital were EBF at hospital discharge (*p* < 0.001).^15^

Two retrospective cohort studies reported a statistically significant increase in overall EBF rates at discharge following the implementation of DHM in their hospitals.^33,35^ Alissa et al. ^35^ reported an increase in yearly average EBF rate from 25% pre-DHM implementation (95% confidence interval [CI] 23.5%–26.5%) to 45.9% post-DHM implementation (95%CI 44.3%- 47.6%, *p* < 0.0001). Merjaneh et al. ^33^ reported an increase in average monthly EBF rates at discharge from 33% pre- to 46% post-implementation of DHM (*p* < 0.005) but did not report confidence intervals or standard deviation. Both studies report data as an overall average percentage of infants who were EBF at discharge, before and after the implementation of DHM and do not report the outcome in the post-DHM implementation cohort separately for infants receiving DHM versus IF supplementation. Thus, the post-DHM implementation group could include both infants who were supplemented with DHM and IF.

The cohort study by Heizelman et al. ^32^ found no difference in feeding type at hospital discharge between DHM and IF supplementation groups. The cohort study by Ponnapakkam et al. ^36^ reported an increase in monthly EBF rates at discharge from 33% (95% CI 0–45%) to 55% (95% CI 30–80%) following the introduction of DHM (magnitude of effect estimated from figures); however, rates of breastfeeding at discharge in the post-DHM implementation cohort were not reported by type of supplemental feeding. As there is an overlap in the confidence intervals of pre- and post-DHM supplementation in figures, this difference is likely not statistically significant.

Overall, we judged the direction of findings as of no clear difference between DHM and IF supplementation on EBF rates at hospital discharge (Table 2). Using the GRADE approach, the certainty of evidence for this outcome was judged as very low due to risk of bias, imprecision, inconsistency, and indirectness (Table 3).

**Table 3:** GRADE Summary of findings (strength of recommendation).

Outcomes	Anticipated absolute effects (95% CI)		Mean difference (95% CI)	N of participants (and N studies)	Certainty of evidence (GRADE)	Comment
	Risk with no DHM	Risk with DHM				
Exclusive breastfeeding at discharge	-	-		*N* = 1966 (5 studies)	Very low	Very low quality^1^ due to: risk of bias; imprecision; inconsistency, and indirectness.
Exclusive breastfeeding after discharge	-	-		*N* = 1703 (2 studies)	Very low	Very low quality^2^ due to: risk of bias; imprecision, and indirectness
Growth (weight, length and head circumference z-scores at discharge)	-	-		*N* = 32 (1 study)	Low	Low quality^3^ due to: risk of bias and imprecision
Duration of hospital stay (days)	2.86	2.95	0.09 (−0.32, 0.50)	*N* = 156 (1 study)	Very low	Very low quality^4^ due to: imprecision and indirectness

^1^*Risk of bias*: limited information concerning study design; *imprecision*: lacking statistical information; *indirectness*: no direct comparison of DHM to IF supplementation group.

^2^*Risk of bias*: lack of blinding & attrition; *imprecision*: small sample size; *indirectness*: different time points for outcomes.

^3^*Risk of bias*: Lack of blinding; *imprecision*: small sample size.

^4^*Imprecision*: small sample size; *indirectness*: inclusion of both infants receiving IF & DHM in comparison group.

GRADE Working Group grades of evidence. High certainty: we are very confident that the true effect lies close to that of the estimate of the effect. Moderate certainty: we are moderately confident in the effect estimate: the true effect is likely to be close to the estimate of the effect, but there is a possibility that it is substantially different. Low certainty: our confidence in the effect estimate is limited: the true effect may be substantially different from the estimate of the effect. Very low certainty: we have very little confidence in the effect estimate: the true effect is likely to be substantially different from the estimate of effect.

##### Exclusive breastfeeding after hospital discharge

Three studies reported effects of supplementation of MOM with DHM compared to IF on EBF after hospital discharge.^33,39^ The pilot RCT by Pithia et al. ^31^ reported similar rates of EBF at 6–8 weeks chronological age in both groups (31% in DHM group and 38% in IF group, *p* = 0.7). The cross-sectional study by Ikonen et al. ^39^ reported higher rates of EBF in infants supplemented with DHM compared to those supplemented with IF at 0–1 month (61.1% *versus* 52.3%, *p* = 0.2), 2–3 months (60.6% *versus* 45.1%, *p* = 0.2) and 4–5 months (40.2% *versus* 21.9%, *p* = 0.001).^39^

Merjaneh et al. ^33^ reported rates of EBF at < 1 month, 4 months, 4−6 months and ≥ 6 months; however, no group or pair-wise comparison were reported. While EBF at < 1 month was lower in the DHM group compared to the IF group (12% versus 67%, respectively), no direct comparison or p-value was given. The authors reported that EBF at ≥ 6 months was higher in the DHM than the IF group (58% vs 15%, respectively) and infants who had received DHM were five times more likely to be EBF at 6 months of life than those who had received IF (adjusted odds ratio [OR] at 6 months = 5.13, 95% CI 1.37– 19.23, *p* = 0.01).^33^ However, there was significant attrition in the IF group (33% versus 46% attrition for DHM and IF groups, respectively).

The available evidence suggests that there was a potential benefit of supplementation of MOM with DHM compared to IF for EBF at 4-6 months, but also no difference for EBF rates up to 3 months of age. Thus, we judged the overall direction of findings as of no clear difference between DHM and IF supplementation on EBF rates after hospital discharge (Table 2). Using the GRADE approach, the certainty of evidence for this outcome was judged as very low due to risk of bias, imprecision, and indirectness (Table 3).

#### Secondary outcomes

##### Any breastfeeding at hospital discharge

One retrospective study reported data on any breastfeeding at hospital discharge,^15^ finding that infants supplemented with IF were significantly less likely to be receiving any breastfeeding at hospital discharge compared to infants supplemented with DHM (Risk ratio 0.84, 95% CI 0.77–0.92). We therefore judged the direction of findings as of possible benefit of DHM on the rates of any breastfeeding at hospital discharge (Table 2).

##### Any breastfeeding after hospital discharge

Four studies reported data on any breastfeeding after hospital discharge.^37–39^ Gray et al. ^37^ showed significantly lower rates of breastfeeding at 2 and 6 months postpartum in infants who received IF supplementation in hospital compared to infants who received DHM supplementation (2 months: OR 0.26, 95% CI 0.12−0.56, p = 0.01; 6 months: OR 0.42, 95% CI 0.19−0.94, *p* = 0.034). Ikonen et al. ^39^ reported different results according to the time point of outcome measurement. While any breastfeeding rates tended to be higher in the DHM compared to IF supplementation group throughout the first year, this was only statistically significant at 4–5 months (87.4% *versus* 68.4%, *p* < 0.001). Riley et al. ^38^ found that rates of breastfeeding were similar between DHM and IF groups at 1 month postpartum. The pilot RCT by Pithia et al. ^31^ reported similar rates of infants received a mixed diet (IF and breastmilk) at 6-8 weeks chronological age, with 44% in the DHM supplementation group and 50% in the IF group (*p* = 0.7).

The effect of DHM supplementation compared to IF on the rates of any breastfeeding after discharge was only significant at some time points and was inconsistent across the three studies reporting this outcome. Thus, we judged the overall direction of findings as of no clear difference between DHM and IF supplementation on any breastfeeding rates after hospital discharge (Table 2).

##### Hypoglycaemia

Three studies reported outcomes related to hypoglycemia using various definitions.^32,34,36^ Sen et al. report median change in infants’ blood glucose concentration following buccal dextrose gel plus a DHM or IF feed and found greater blood glucose concentration in infants supplemented with DHM compared to those supplemented with IF, but this was not statistically significant.^34^ Heizelman investigated rates of hypoglycemia resolution with a single dose of dextrose gel and the number of additional doses required to resolve hypoglycemia and found no difference between study groups.^32^

Ponnapakkam et al. reported a significant increase in blood glucose concentration following a feed of expressed breastmilk plus supplementation with DHM compared to supplementation with IF (mean (95% CI) increase in blood glucose with DHM: 20.7 (12.7–28.7) mg/dL *vs*. with IF: 6.6 (2.5–10.7) mg/dL). As there was no overlap in reported confidence intervals, we concluded that the difference reported was likely statistically significant. In addition, this study reports that episodes of symptomatic hypoglycemia remained unchanged following the introduction of DHM in their unit and that time to final hypoglycemic episodes was similar between DHM and IF supplementation.^36^

Thus, the direction of findings was judged as of no clear difference between DHM and IF supplementation for outcomes relating to hypoglycemia (Table 2).

##### Infant growth

One study reported outcomes related to infant growth, with no significant difference in mean (SD) weight, length, and head circumference (HC) z-scores between DHM and IF supplementation groups at the end of study intervention.^31^ At 6–8 weeks chronological age, there was no difference in weight and length of both groups except for higher HC z-score in the IF group (DHM −0.4 (1.3) vs. IF 0.4 (0.9), p = 0.04). Since it was a pilot RCT with small sample size, we judged the overall direction of findings for this outcome as of no clear difference (Table 2). Using the GRADE approach, the certainty of evidence for this outcome was judged as low due to risk of bias and imprecision (Table 3).

##### Admission to NICU and duration of hospital stay

Ponnapakkam et al. ^36^ described a reduction in NICU admission rates for treatment of asymptomatic hypoglycaemia from 16% (95% CI 0-38%) in the pre-DHM implementation cohort to 6% (95% CI 0-28%) following the implementation of DHM (confidence intervals estimated from figure). This difference is unlikely to be significant.^36^ Heizelman et al. found no significant difference in NICU admission rates between DHM and IF supplementation groups; however, it was not reported whether this outcome was a measure of all-cause admission or specifically related to hypoglycemia.^32^ Mannel et al. ^15^ reported that length of hospital stay did not differ between study groups.^15^

Therefore, the overall direction of findings was judged as of no clear difference between DHM and IF supplementation on the rates of admission to NICU and duration of hospital stay (Table 2). Using the GRADE approach, the certainty of evidence for this outcome was judged as very low due to imprecision and indirectness (Table 3).

##### Childhood morbidity

The study by Saarinen et al. ^30^ was the only study reporting on childhood morbidity, defined by the authors as the risk of cow’s milk allergy (CMA) during childhood. This RCT found that there was no difference in risk of developing CMA between infants supplemented during hospital stay with cow’s milk-based IF and with DHM. Therefore, the direction of findings was judged to be of no clear difference between DHM and IF supplementation on childhood morbidity (Table 2).

### Quality assessment and risk of bias

**Fig. 2:**
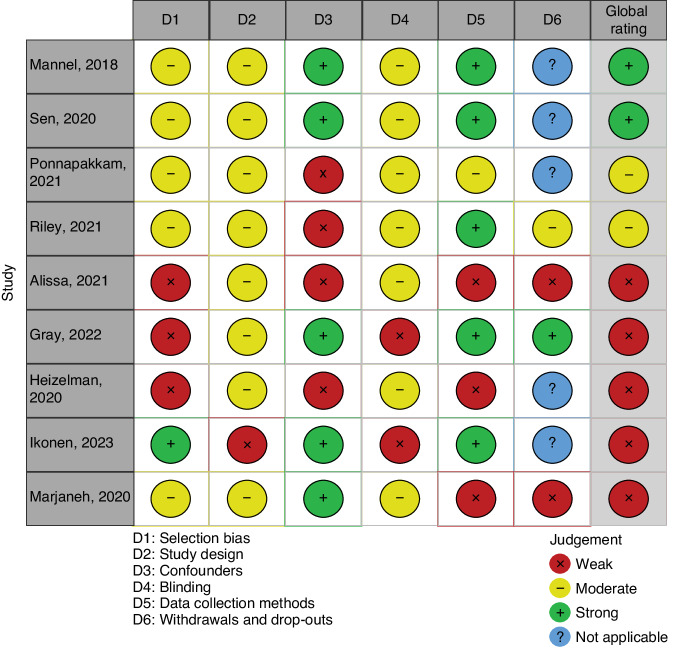
Quality assessment of observational studies. Quality assessment of observational studies across six domains assessed as weak (red), moderate (yellow), strong (green) or not applicable (blue) using the EPHPP Quality Assessment Tool.

Among the nine observational studies, we judged five studies to be of weak quality,^32,33,35,37,39^ two of moderate quality^36,38^ and two of strong quality^15,34^ (Fig. 2).

Of the five studies assessed as being of weak quality, four were cohort studies,^32,33,35,37^ and one was a cross-sectional study.^39^ The quality of these studies was further downgraded due to: potential for selection bias^37,38,40,41^; failure to control for confounders^37,40^; study design^37,38,40–42^; lack or poor description of blinding^37,38,40–42^; data collection methodology,^37,38,40^ and attrition.^38,40^

The two studies assessed as of moderate quality were prospective cohort studies.^36,38^ The quality of these studies was further downgraded due to: lack of control for potential confounders^43,44^; selection bias^43,44^; lack or poor description of blinding^43,44^; data collection methodology,^43^ and attrition.^44^

Two studies were assessed as being of strong quality^15,34^ ; but since both are retrospective cohort studies, they were judged as of moderate quality in the domains of study design, selection bias and blinding. The intervention integrity was judged uncertain within the study by Sen et al. as the authors do not clearly state whether infants were receiving IF or DHM exclusively or as supplementation to breastfeeding.

**Fig. 3:**
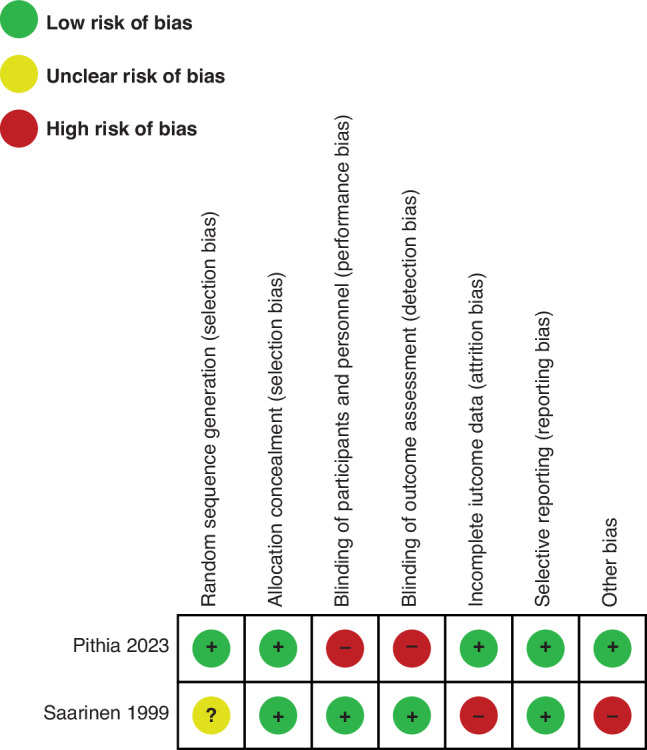
Risk of bias for included randomized controlled trial. Risk of bias for included randomized controlled trials across seven domains was assessed as low (green), unclear (yellow) or high (red) using the RoB1 Tool.

The quality of the two included RCTs^30^ was assessed using the ROB-1 tool (Fig. 3). The study by Saarinen et al. ^35^ was judged to be at low risk of reporting bias due to adequate blinding and concealment of allocation of the intervention from participants, clinicians and outcomes assessors. This study was judged as of unclear risk of bias for random sequence generation as the method was not clearly described, and as high risk of bias for incomplete outcome reporting and other biases. Outcome data were considered as inadequately addressed because, although losses to follow-up were low (0.9%), only 76% of the participants returned completed records of infant feeding regimens, and detection of the primary outcome (diagnosis of CMA) was reliant on parental reporting of infant’s symptoms. Authors assumed that if symptoms were not reported, they were not present; however, it is possible that symptoms were not reported due to attrition. Other biases include selection bias, as baseline characteristics were not described and only 41% of the eligible population agreed to participate in the study, and a potential conflict of interest as this study received funding from industry (Valio Limited, a dairy product manufacturer in Finland; and Nutricia, a brand of Danone specialising in therapeutic food and IF).

The pilot RCT by Pithia et al. ^31^ was judged to be of low risk of bias in the domains of selective reporting bias, adequate sequence generation, allocation concealment and incomplete outcome data. This study was judged as high risk of bias regarding blinding, as this RCT was unblinded to participants, clinicians and outcome assessors. No other biases were identified.

## Discussion

This systematic review evaluated the effect of supplementation of MOM with DHM compared to IF in MLP and ET infants. Findings from a small number of studies were conflicting and, due to significant heterogeneity in outcome definitions, meta-analysis was not possible. To the best of our knowledge, this is the first systematic review to compare the effects of DHM to IF supplementation in this population, and six ongoing RCTs were identified.

The majority of included studies were observational, with only two RCTs identified; thus, the quality of evidence was assessed as predominantly weak, with only two observational studies assessed as being of high quality.^15,34^ Most limitations were methodological issues, including failure to control confounders,^32,35,36,38^ inadequate description of or lack of blinding,^37,39^ selection bias,^32,35,37^ and attrition.^33,35^ Some of the included studies provided poor descriptions of study groups,^15^ and none of the included studies reported outcomes stratified by GA, making it difficult to determine the benefits and risks for MLP and ET infants separately. Most studies also included FT infants.^30,32–34,36–39^ One RCT identified in this review was assessed as being of high risk of bias,^30^ while the other was assessed as being of low risk of bias.^31^ In addition, the certainty of evidence for four pre-specified GRADE outcomes was judged to be low or very low.

The increasing acceptance and use of DHM in healthy infants in hospitals and the community (through informal milk sharing), identified by McCune et al. ^40^ highlights the need for high-quality, controlled trials examining the impact of DHM supplementation on breastfeeding rates and other health outcomes such as growth, childhood morbidity and neurodevelopment. A systematic review by Williams et al. ^41^ showed improvements in any breastfeeding rates at discharge when DHM was introduced to neonatal units, but not EBF rates. Similar to our review, they did not find evidence that DHM use had an adverse effect on breastfeeding rates. Furthermore, DHM used in hospital settings is often pasteurized while informal milk sharing in the community often consists of the provision of unpasteurized DHM to healthy infants, and whether this poses any health risk to infants should be further investigated.

The impact of DHM supplementation on EBF rates at discharge remains unclear. This finding is consistent with McCune et al. ^40^ who also found conflicting evidence. The added cost of providing DHM over IF, calculated to be 38 times higher in a Canadian clinical trial,^43^ also highlights that the benefits of this intervention must be thoroughly examined. Displacement of MOM by introducing breastmilk substitutes (DHM or IF) has been hypothesised to lead to decreased breastmilk production.^44^ However, we found no evidence to indicate that providing DHM instead of IF supplementation may negatively impact breastfeeding. Nevertheless, considering the many benefits provided by EBF on decreased short and long-term health morbidities,^8^ improved lactation support must be prioritized for mothers of MLP and ET infants to achieve successful breastfeeding.^10^

Outcome measures varied between studies. While most included studies reported breastfeeding rates,^15,32,33,35–39^ this varied both in how it was defined (any *versus* exclusive) and time point at which it was measured (hospital discharge, one month and up to 12 months). In addition, it is worth noting that some preterm infants might be discharged from hospital receiving exclusive MOM feeds via both breast and bottle, which might not be classified as EBF. In view of this heterogeneity, the effect of DHM supplementation on total duration of breastfeeding remains unclear.

Most studies in this review did not include multiples and they were explicitly excluded from two studies.^38,39^ The risk of preterm birth is significantly higher in multiple compared to single pregnancies, particularly for MLP births.^42^ Research from Iceland, a country with one of the highest rates of breastfeeding, suggests that twins born late preterm may struggle to continue any breastfeeding beyond one month compared with twins born full-term.^45^ Given the higher incidence of preterm birth and subsequent breastfeeding challenges in this population, often requiring supplementation of MOM, multiples should be included in future studies investigating the effects of supplementation of MOM with DHM compared to IF.

The effect of DHM supplementation compared to IF on hypoglycemia was highly variable regarding the intervention provided and the definition of outcome measures.^32,34,36^ In two studies, the intervention consisted of the provision of dextrose gel plus a supplemental feed with DHM or IF,^32,34^ whereas the third excluded infants who had received dextrose gel.^36^ This may reflect a general variation in practice regarding the treatment of neonatal hypoglycemia.

The recommended first-line treatment for neonatal hypoglycemia, especially in asymptomatic cases, combines dextrose gel and feeding.^46^ McCune et al. found one of the commonly cited reasons for increasing DHM usage was for the treatment of neonatal hypoglycemia, with one study showing 73% of parents preferred DHM over IF for this indication.^40^ This demonstrates that when available, DHM is likely an acceptable alternative for treating neonatal hypoglycemia, despite unclear evidence of any benefits. As demonstrated in this review, it remains unclear whether supplementation of MOM with DHM results in better glycemic control compared to IF supplementation.

The Glucose in Well Babies Study (GLOW study),^47^ found that feeding with IF may result in greater increases in interstitial glucose concentration than breastfeeding, although a limited number of infants receiving IF or DHM hindered the comparison. DHM often consists of mature human milk (produced after 2 weeks postpartum), but the composition varies widely and, in many cases, the mean energy, protein and fat content are below the reference range of preterm human milk.^48^ Nevertheless, mature milk has higher energy density than colostrum, which is lower in fat and lactose.^49^ As the composition of most IF is based on the nutritional composition of mature milk, IF tends to be higher in energy, carbohydrates and fat than colostrum, which may lead to greater increases in interstitial glucose. The studies included in this systematic review did not report the nutritional composition of DHM. Future studies should report the nutritional composition of DHM and volume ingested so that any correlations between glycemic response and nutritional intake can be determined.

Only one of the studies included in this review examined growth, with unclear effects of supplementation of MOM with DHM compared to IF.^31^ A recent systematic review including very to moderate preterm infants suggested that increasing DHM usage was associated with a decrease in daily weight gain and head growth compared to both breastfed and formula-fed infants.^50^ This is similar to the effect suggested by Quigley et al. ^17^ however, only a few RCTs were included in this systematic review. To better assess potential long-term risks or benefits of using DHM instead of IF for supplementation of MOM, future research should report growth in a standardised way.

### Limitations & strengths of the review

This review has several strengths. A systematic, comprehensive literature search was performed using multiple databases, and not limited by date or language of publication. Randomized and observational studies were included, and a wide range of outcomes of interest for low-risk infants were reviewed for the first time. Our protocol was prospectively registered in Prospero with predefined criteria, therefore limiting the potential for reporting bias based on the identified evidence. The selection of studies, data extraction, and assessment of quality and risk of bias were carried out independently by two researchers, and disagreements mediated by a third reviewer. Quality assessment and risk of bias were assessed using validated tools,^24,26^ and the overall certainty of evidence was assessed using the GRADE tool.^26^

Nonetheless, this review has some limitations. Pooling of data for meta-analysis was not possible due to significant heterogeneity of population, intervention, comparator, outcome assessment and reporting. Two of the included studies were only available as abstracts^32,35^ and provided limited data on outcomes of interest. No study reported results stratified by GA and, while the ET and late preterm infants were well represented, few included moderate preterm infants; therefore, findings cannot be generalised to this population. All included studies were from the United States or Finland, and one of the RCTs was conducted over 20 years ago; since then, neonatal care has significantly improved. This highlights the need for more contemporary studies to be conducted, including populations with limited access to DHM banks and in resource-limited settings that are more impacted by the burden of prematurity.^1,51^

## Conclusion

This review found no clear evidence of the effect of the supplementation of MOM with DHM compared to IF for MLP and ET infants on a range of health outcomes, including rates of EBF. Limitations in access to human milk banks, heterogeneity in definition of study population and outcomes, and scarcity of high-quality studies in this population limited the evidence available for this review. With the increasing popularity of DHM supplementation and proliferation of human milk banks and informal milk sharing, further high-quality studies are needed to assess the effects of DHM supplementation on health outcomes in MLP and ET infants.

## Supplementary information

PRISMA_checklist_SR DHM vs IF

Supplementary tables

## Data Availability

Data will be made available upon reasonable request submitted to corresponding author.
